# Survival of Spoilage and Pathogenic Microorganisms on Cardboard and Plastic Packaging Materials

**DOI:** 10.3389/fmicb.2017.02606

**Published:** 2017-12-22

**Authors:** Lorenzo Siroli, Francesca Patrignani, Diana I. Serrazanetti, Cristiana Chiavari, Marzia Benevelli, Luigi Grazia, Rosalba Lanciotti

**Affiliations:** ^1^Department of Agricultural and Food Sciences, Alma Mater Studiorum, University of Bologna, Campus of Food Science, Cesena, Italy; ^2^Interdepartmental Center for Industrial Agri-Food Research, Alma Mater Studiorum, University of Bologna, Cesena, Italy; ^3^Department of Agricultural and Food Sciences, Alma Mater Studiorum, University of Bologna, Reggio Emilia, Italy

**Keywords:** entrapping, pathogens, safety, cardboard, plastic, scanning electron microscopy

## Abstract

The aim of this work was to study the interaction of corrugated and plastic materials with pathogenic and spoiling microorganisms frequently associated to fresh produce. The effect of the two packaging materials on the survival during the storage of microorganisms belonging to the species *Escherichia coli*, *Listeria monocytogenes*, *Salmonella enteritidis*, *Saccharomyces cerevisiae*, *Lactobacillus plantarum*, *Pseudomonas fluorescens*, and *Aspergillus flavus* was studied through traditional plate counting and scanning electron microscopy (SEM). The results obtained showed that cardboard materials, if correctly stored, reduced the potential of packaging to cross-contaminate food due to a faster viability loss by spoilage and pathogenic microorganisms compared to the plastic ones. In fact, the cell loads of the pathogenic species considered decreased over time independently on the inoculation level and packaging material used. However, the superficial viability losses were significantly faster in cardboard compared to plastic materials. The same behavior was observed for the spoilage microorganisms considered. The SEM microphotographs indicate that the reduction of superficial contamination on cardboard surfaces was due to the entrapping of the microbial cells within the fibers and the pores of this material. In addition, SEM data showed that the entrapped cells were subjected to more or less rapid lyses, depending on the species, due to the absence of water and nutrients, with the exception of molds. The latter spoilers were able to proliferate inside the cardboard fibers only when the absorption of water was not prevented during the storage. In conclusion, the findings of this work showed the reduction of cross-contamination potential of corrugated compared to plastic packaging materials used in fruit and vegetable sector. However, the findings outlined the importance of hygiene and low humidity during cardboard storage to prevent the mold growth on packaging.

## Introduction

In the last few years, various food-borne illnesses have been attributed to the consumption of fresh vegetables and fruits. Norovirus, *Salmonella* spp., *Listeria monocytogenes*, and *Escherichia coli* O157:H7 are reported to be the leading cause of fresh produces related outbreaks (Alegre et al., 2010; Scallan et al., 2011; Oliveira et al., 2012; Siroli et al., 2014; Callejón et al., 2015). Pathogenic and spoilage microorganisms can contaminate fresh products in different phases of the product processing and, given the absence of treatments that can eliminate microorganisms, can reach the end consumer (Francis et al., 2012). Moreover, the maintaining of refrigerated temperatures throughout the whole supply chain is often very difficult and thermal abuses can lead to an increase of microbial growth rates both on the packaging surfaces and the products (Kusumaningrum et al., 2003). In addition, the long-lasting permanence of some microorganisms on the package surface of fresh produces can lead to their rapid multiplication and possible biofilm formation (Barnes et al., 1999; Uhlich et al., 2006; Wilks et al., 2006; Shi and Zhu, 2009). The presence of biofilm can provide protection to pathogenic and spoilage microorganisms that can survive longer and be involved in cross-contamination phenomena from packaging to food (Bae et al., 2012; Valeriano et al., 2012). The packaging of fresh products, and in particular its microbiological quality, can play a very important role both in the quality and safety of the product. Very limited and fragmentary information regarding the microbial cell loads present on the surfaces of packaging materials are available in literature. Nevertheless, the presence of pathogens and spoilage microorganisms on the packaging surfaces is documented (Suominen et al., 1997; Mafu et al., 2011; Ismaïl et al., 2013). Differences in chemical/physical characteristics, correct storage, and sanitizing of packing materials strongly affect the microbial loads present on the packaging surfaces. In fact, reusable packaging generally presents aerobic mesophilic loads between 3 and 6 log CFU/cm^2^, while virgin fiber packaging presents cell loads ranging between 2 and 5 log CFU/cm^2^. Several microorganisms were detected on the packaging surfaces and spore forming bacteria such as *Bacillus* spp. and *Clostridium* spp. and molds such as *Aspergillus* spp. and *Cladosporium* spp. are the more involved (Suominen et al., 1997; Binderup et al., 2002; Turtoi and Nicolau, 2007; Patrignani et al., 2016). Nevertheless, the presence of pathogenic microorganisms in not well-sanitized packaging material is reported in literature (Patrignani et al., 2016). Given this situation it is important to select an appropriate packaging based on the product characteristics and to prevent possible microbial contamination (Campos et al., 2014). Corrugated cardboard packaging is one of the most widely used types for packaging fresh products and presents significant advantages from an environmental and microbiological standpoint than plastic materials (Levi et al., 2011; Hladikova et al., 2015).

As things stand at present, it is extremely complicated to determine how and under what role the packaging can contribute to microbial cross-contamination of the packaged product. In fact, the microbial presence on packaging surfaces as well as on packed fruits and vegetables is difficult to establish because it has a very high variability (Siroli et al., 2014; Patrignani et al., 2016). Additionally, the storage conditions such as temperature and relative humidity of the packed products, and the eventual presence of biofilms and availability of nutrients play a key role in the survival and multiplication of microbial communities, affecting the possibility of cross-contamination from the package to the fresh product (Siroli et al., 2014; De Candia et al., 2015). However, cross-contamination phenomena from packaging to the product are also influenced by the characteristics of the packed fruit or vegetable as the degree of ripeness, acidity, sugar content, and presence of physical damages (Heaton and Jones, 2008).

Recently Patrignani et al. (2016) demonstrated, trough challenge tests and a modeling approach, a higher transferring of the microorganisms *E. coli*, *Pseudomonas* spp., and *Saccharomyces cerevisiae* in peaches packed in reusable plastic containers (RPC) compared to corrugated cardboard. The authors demonstrated a reduction of potential cross-conta mination from packaging to fruits in cardboard compared to plastic as well a higher microbiological quality of peaches stored in cardboard boxes. These authors hypothesized that the better microbial qualities and the reduction of cross-contamination of cardboard compared to RPC were due to the capability of cardboard to entrap microbial cells. Also Brandwein et al. (2016) studied the relationship between corrugated cardboard packaging and fresh produce and the potential of an anti-biofilm polymer to coat corrugated cardboard surfaces in order to mitigate surface bacterial biofilm formation. However, to our knowledge, the literature data on this topic are still scarce. Thus, this research was aimed to study the interaction of corrugated and plastic materials with different microbial species, such as pathogens (*L. monocytogenes*, *E. coli*, *Salmonella enteritidis*) and spoiling (*S. cerevisiae*, *Lactobacillus plantarum*, *Pseudomonas fluorescens*, *Aspergillus flavus*) ones. In particular, the effect of the two packaging materials on the survival during the storage of the target microorganisms was studied through traditional plate counting and scanning electron microscopy (SEM). To reach the research goals, the target microorganisms were inoculated, at the same levels, on corrugated and plastic surfaces and their microbial cell loads were monitored over time. In addition, to understand the fate of the inoculated microorganisms, the packaging material sections were studied by SEM.

## Materials and Methods

### Microbial Strains

The microbial strains employed in this work were *E. coli* 555, *L. monocytogenes* SCOTT A, *S. enteritidis* E5, *S. cerevisiae* spa, *Lb. plantarum* 82, *P. fluorescens* 4T04, and *A. flavus* belonging to the Department of Agricultural and Food Sciences of Bologna University.

*Escherichia coli* 555, *L. monocytogenes* SCOTT A, *S. enteritidis* E5, and *P. fluorescens* 4T04 were preliminarily grown on Brain Heart Infusion (BHI) Broth (Oxoid Ltd., Basingstoke, United Kingdom) and incubated at 37°C for 24 h. *S. cerevisiae* and *A. flavus* were grown in Yeast Extract Peptone Dextrose (YPD) broth (Oxoid Ltd., Basingstoke, United Kingdom) at 25°C for 48 h and *Lb. plantarum* 82 was grown on De Man, Rogosa, and Sharpe (MRS) broth (Oxoid Ltd., Basingstoke, United Kingdom) at 37°C for 24 h.

### Entrapping Capability of Corrugated Compared to Plastic

Corrugated and plastic surfaces were used for studying the entrapping capability of each material. The size of the surfaces was 5 cm^2^ both for corrugated and plastic.

The target microorganisms, pre-cultivated as reported in the section “Microbial Strains,” and refreshed overnight in the appropriate growth media were inoculated in the considered surfaces separately and at three different inoculum levels (ranging between 2.0 and 6.0 log CFU/cm^2^). The microorganisms were prepared by making serial dilutions in sterile physiological solution (0.9% NaCl). The 5 cm^2^ surfaces were inoculated with 500 μl of the appropriate dilution. The inoculated surfaces were dried at room temperature (25°C) for 30 min and sampled after 1, 8, 24, and 48 h. To recover the microorganisms, a swabbing method (Ismaïl et al., 2013) was adopted by using a classic hygienic cotton swab moistened in sterilized peptone water 0.1% (Oxoid Ltd., Basingstoke, United Kingdom). Then the swab was immersed in 10 ml of maximum recovery diluent (MRD) (Oxoid Ltd., Basingstoke, United Kingdom) and if necessary serial dilution in MRD broth was performed. Finally, the microorganisms were enumerated in selective media. In particular, Listeria Selective Agar Base (Oxoid Ltd., Basingstoke, United Kingdom) was used for the enumeration of *Listeria*, Violet Red Bile Agar (Oxoid Ltd., Basingstoke, United Kingdom) for *E. coli*, Bismuth Sulfite Agar (Oxoid Ltd., Basingstoke, United Kingdom) for the detection of *Salmonella*, Pseudomonas Agar Base (Oxoid Ltd., Basingstoke, United Kingdom) for *Pseudomonas*, YPD for the enumeration of *S. cerevisiae*, and MRS (Oxoid Ltd., Basingstoke, United Kingdom) for the enumeration of *Lb. plantarum*. For each analysis, three independent repetitions were performed in different days.

### Scanning Electron Microscopy (SEM) Analyses

The SEM analyses were performed on corrugated surfaces, inoculated separately with at a level of 6 log CFU/cm^2^ with *L. monocytogenes*, *E. coli*, *S. cerevisiae* and *Lb. plantarum*, and *A. flavus* after 1, 24, and 48 h. Moreover, a trial on corrugated surfaces inoculated with a mixture of all the target microorganisms was performed. Furthermore, analyses on plastic surfaces not inoculated and inoculated with *Lb. plantarum* were performed.

Relative to bacteria and yeasts, the method suggested by Sowden and Walker (1988) was applied, introducing modifications concerning the preparation of individual samples.

After the inoculations small pieces of packaging were cut out with a razor blade and dehydrated stepwise in ethanol (50–100%) at room temperature after critical point the specimens were mounted on SEM discs, coated with golds, and observed using a Hitachi S-510 SEM (Hitachi, Tokyo, Japan).

For *A. flavus* observation, due to the mold fragility, the protocol suggested by Edelmann and Klomparens (1994) was employed. The samples were firstly fixed with glutaraldehyde and then dehydrated with the graded ethanol series (50–100%).

### Statistical Analysis

Microbiological data were examined with STATISTICA software v. 8.0 (TIBCO Statistica, Palo Alto, CA, United States) and a two way-ANOVA followed by Fisher’s least significant difference (LSD) test at *p* < 0.05 were performed.

## Results and Discussion

### Cell Loads of Spoilage Microorganisms in Relation to Inoculation Level, Packaging Material, and Storage Time

**FIGURE 1:**
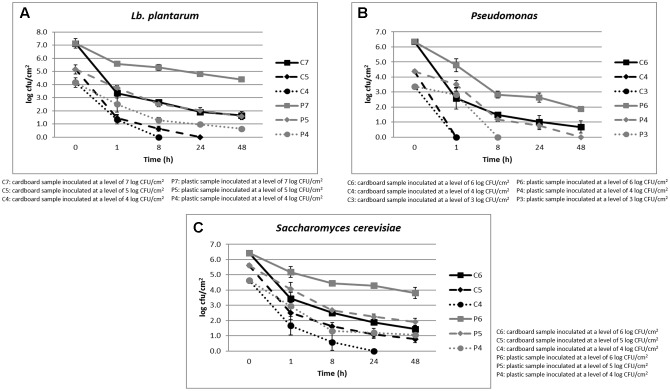
**(A–C)** Cell loads recorded during the storage at environmental temperature of *Lb. plantarum* **(A)**, *P. fluorescens* **(B)**, and *S. cerevisiae* **(C)** in relation to the inoculation levels and the packaging materials.

The cell loads recorded during the storage at environmental temperature of *Lb. plantarum* in relation to the inoculation levels (ranging between 4 and 7 log CFU/cm^2^) and the packaging materials are shown in **Figure 1A**. Its cell load decreased over time independently on the inoculation level and packaging materials. However, the decrease was significantly faster in cardboard compared to plastic materials. In fact, after 24 h of storage, the *Lb. plantarum* cell load decreases were higher than 5 log CFU/cm^2^ in cardboard and less than 3 log CFU/cm^2^ in plastic material. In addition, on cardboard material, when the inoculation levels were of 4–5 log CFU/cm^2^, *Lb. plantarum* resulted under the detection limit (0.5 log CFU/cm^2^) after 8 and 24 h of storing at environmental conditions (25°C), respectively. By contrast, for plastic material, also considering the lowest inoculation level, the *Lb. plantarum* cell loads never decreased under the detection limits.

The results obtained for *P. fluorescens*, during the storage at environmental temperature, in relation to the cell loads inoculated (ranging between 3 and 6 log CFU/cm^2^) and the packaging materials are shown in **Figure 1B**. Also for this spoilage bacteria, the same behavior of *Lb. plantarum* was observed in relation to the packaging material. However, *P. fluorescens* resulted more sensitive to the environmental conditions, decreasing more quickly its viability both on plastic and cardboard materials. In fact, with an inoculation level of 3 log CFU/cm^2^, it reached contamination levels under the detection limit after 1 and 8 h in cardboard and plastic, respectively. When the inoculation level increased to 4 log CFU/cm^2^, *P. fluorescens* dropped under the detection limit after 1 and 48 h in cardboard and plastic materials, respectively.

Among the spoilage microorganisms considered, *S. cerevisiae* resulted the most resistant to the environmental conditions, showing the lowest cell load reductions (**Figure 1C**). In fact, its cell load decreases down to the detection limit were observed after 24 h only on cardboard surfaces and with a contamination level of about 3 log CFU/cm^2^. However, significant differences in terms of *S. cerevisiae* cell loads between plastic and cardboard were observed independently on the inoculation levels and storage times. The reduction of the superficial cell load levels of all the spoilage microorganisms inoculated on the surfaces of the packaging materials clearly indicated the decrease of the contamination potential of cardboard compared to plastic material of packed fruit. The reduction of spoilage microorganism cross-contamination due to the packaging material is considered fundamental to increase the fruit shelf-life and quality. These results are in line with the outcomes obtained by Patrignani et al. (2016) that showed a reduction of potential cross-contamination by pathogens and spoiling microorganisms from packaging surfaces to fruits in cardboard compared to plastic.

### Cell Loads of Pathogenic Microorganisms in Relation to Inoculation Level, Packaging Material, and Storage Time

**FIGURE 2:**
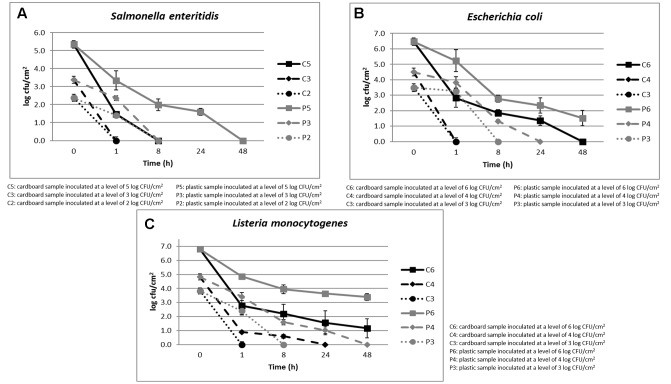
**(A–C)** Cell loads recorded during the storage at environmental temperature of *S. enteritidis* **(A)**, *E. coli* **(B)**, and *L. monocytogenes* **(C)** in relation to the inoculation levels and the packaging materials.

The cell loads recorded during the storage at environmental temperature of *S. enteritidis* in relation to the inoculation levels (ranging between 2 and 5 log CFU/cm^2^) and the packaging materials are shown in **Figure 2A**. The cell loads of this pathogenic species decreased over time independently on the inoculation level and packaging material used. However, the cell load decrease was significantly faster in cardboard compared to plastic materials.

In fact, with inoculation levels of about 2 and 3 log CFU/cm^2^, *S. enteritidis* reached cell loads under the detection limit (1 log CFU/cm^2^) after 1 and 8 h on cardboard and plastic surfaces, respectively. When the inoculation level increased to about 5 log CFU/cm^2^, this microorganism decreased its cell loads under the detection limit after 8 and 48 h in cardboard and plastic surfaces, respectively. Similar results were obtained for *E. coli*, as shown in **Figure 2B**.

As shown in **Figure 2C**, with inoculation level of about 3 log CFU/cm^2^, also *L. monocytogenes*, a dangerous psychotropic Gram-positive food pathogen, decreased its cell load under the detection limit within 1 and 8 h of storage at environmental conditions on cardboard and plastic surfaces, respectively. As expected, this pathogenic species was more resistant to the environmental conditions compared to the Gram-negative species considered (*S. enteritidis*, *E. coli*, and *P. fluorescens*). In any case, the decrease of *L. monocytogenes* cell load was significantly higher on cardboard that on plastic surface. On the other hand, it is widely reported that Gram-positive bacteria and yeasts could survive longer at environmental conditions on inert surfaces (Robine et al., 2000; Tang, 2009).

The reduction of the superficial cell load levels of all the pathogenic microorganisms inoculated on the surfaces of the packaging materials clearly showed the reduction of the contamination risk in fruit packaged in cardboard compared to that packaged in plastic material. The reduction of pathogen cross contamination due to the packaging material is fundamental to increase the fruit safety features and to decrease the occurrence of food borne diseases associated to the intake of fruits and vegetables. The role contact surface in the contamination of food is widely recognized and consequently in Europe ECR 852-2004 regulates and specify the measures that have be carried out to guarantee a safe contact between products and packaging material and to avoid chemical and microbial contamination. For example, there are stringent limits for pathogens in the Annex 1^1^ (Carpentier and Barre, 2012).

### Scanning Electron Microscope Analyses

To understand if the cell load reductions observed were due to the cell viability losses or to the entrapment of the microorganisms within the cardboard fibers, the inoculated surfaces were studied through SEM technique.

**FIGURE 3:**
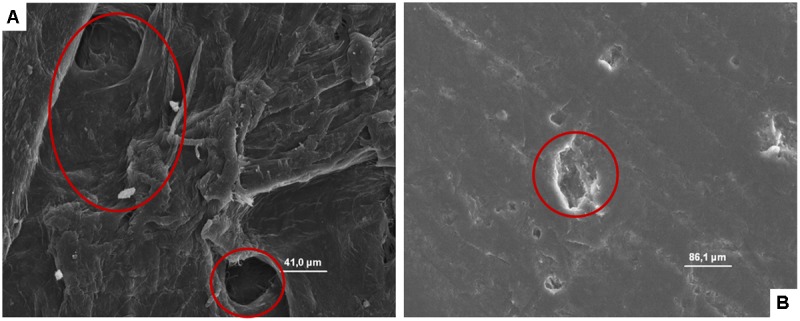
**(A, B)** SEM microphotographs of cardboard **(A)** and plastic **(B)**. In **A** the presence of pores is evident, while in **B** a smooth, plane, and homogeneous surface with the presence of a clear scratch is evident.

The microphotographs obtained showed, independently on the microbial species considered, the great capability of cardboard to entrap the microbial cells contributing in a significant manner to reduce the superficial contamination level in terms of both spoilage and pathogenic microorganisms and, consequently, its role in the contamination of packed fruit with the same microorganisms. In fact, the microstructure of the cardboard showed pores ranging between few to hundreds of microns (**Figure 3A**). Microbial cells considered in these studies have dimensions ranging between 0.7 (*L. monocytogenes*) to about 10 μm (*S. cerevisiae*).

By contrast, plastic material (**Figure 3B**) showed a smooth, plane, and homogeneous surface without hole, and micro-pores able to entrap microorganisms.

On the other hand, it is well known that the microbial contamination of surfaces, the survival of microorganisms and their eventual growth or death rates depend on their features and conditions. For example, characteristics as roughness and porousness or the execution of a sanitization process strongly affect microbial survival (Ismaïl et al., 2013; Montibus et al., 2016). By contrast, on the plastic surfaces (even if new and sanitized very well) were evident cuts and scratches due to the re-usage able to recover in case of inappropriate cleaning and sanitization processes organic matter in which microorganisms are reported to proliferate.

The role of microstructure of packaging materials in the transferring of microorganism to fruits had been recently underlined also by Montibus et al. (2016). These authors showed through a challenge test that poplar crates, being a porous material, guaranteed a reduced cross-contamination *Penicillium expansum* conidia and *E. coli* of packaged apples. These authors showed that *P. expansum* conidia survived but did not grow on wood specimens if the absorption of humidity was prevented. They showed also that *E. coli* decreased its cell loads on the poplar crate surfaces after 1 h from the inoculation. Also, these authors attributed the reduction of superficial cell loads and of the apple contamination potential to the entrapping capability of poplar wood without demonstrating it with SEM analyses. In fact, wood, analogously to corrugated surface is characterized by a high roughness and porousness. For example, poplar has several pores of small size and regularly placed throughout a growth ring (Jacquiot et al., 1973).

**FIGURE 4:**
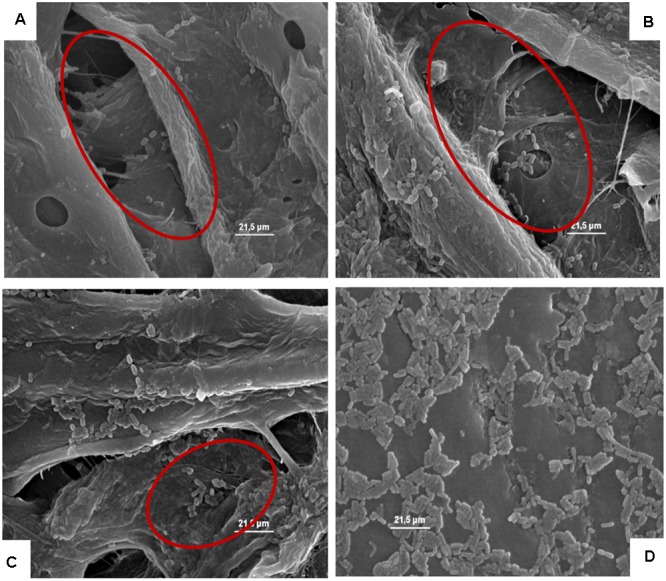
**(A–D)** Presence of the inoculated *Lb. plantarum* on cardboard surface after 1 h **(A)**, 24 h **(B)**, 48 h **(C)**, and after 1 h on plastic surface **(D)**. In **A–C** the presence of microbial cells in pores formed between cardboard fibers is evidenced. In **D**, it is highlighted that all the microbial cells remain all on the surface of the packaging material.

**FIGURE 5:**
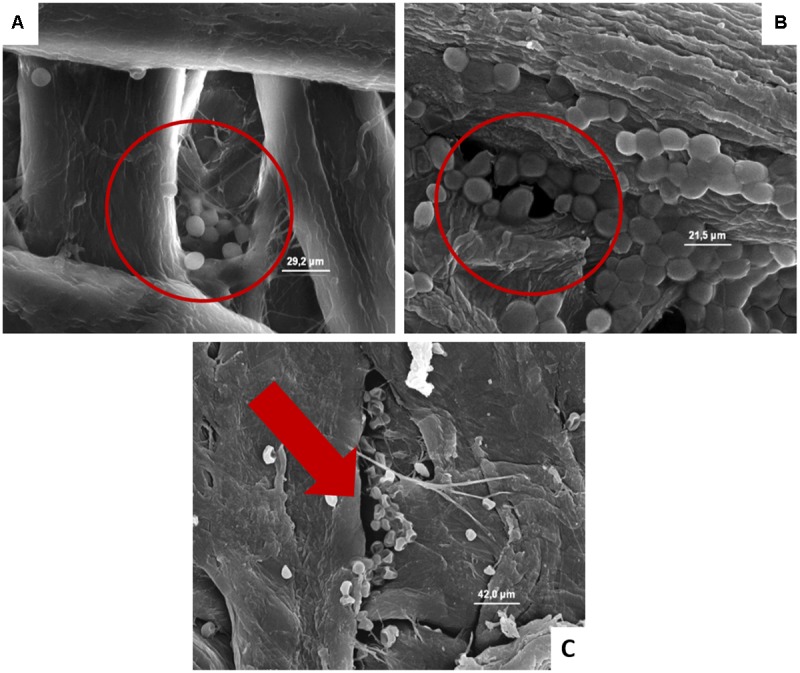
**(A–C)** Presence of the inoculated *S. cerevisiae* on cardboard surface after 1 h **(A)**, 24 h **(B)**, and 48 h **(C)**. In **A** and **B** is evidenced the entrapping of microbial cells in pores formed between cardboard fibers. In **C** the cell lyses also of entrapped cells is outlined by the red harrow.

**FIGURE 6:**
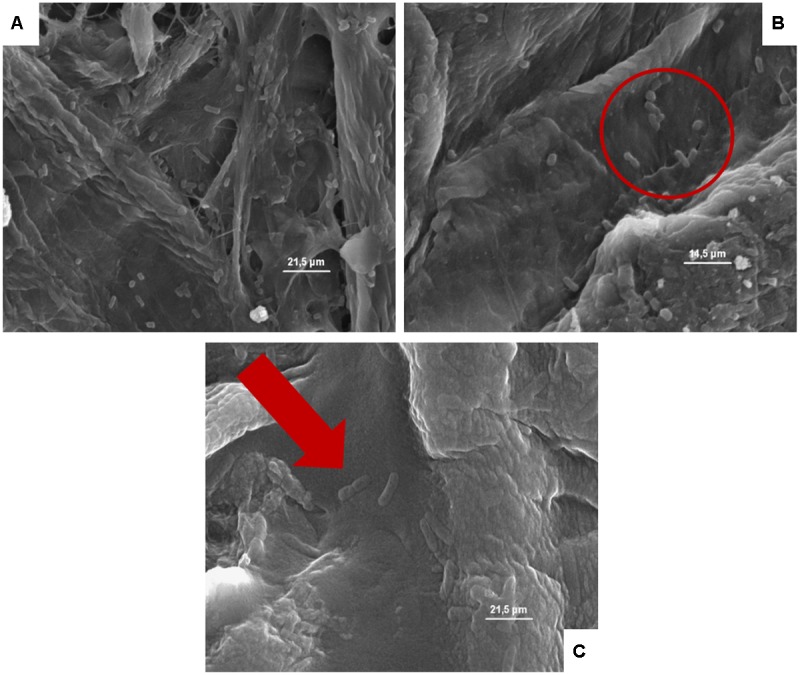
**(A–C)** Presence of the inoculated *Escherichia coli* on cardboard surface after 1 h **(A)**, 24 h **(B)**, and 48 h **(C)**. In **A** and **B** is evidenced the presence of pores and the entrapping of microbial cells between cardboard fibers. In **C**, the *E. coli* lysis also of entrapped cells is outlined by the red harrow.

**FIGURE 7:**
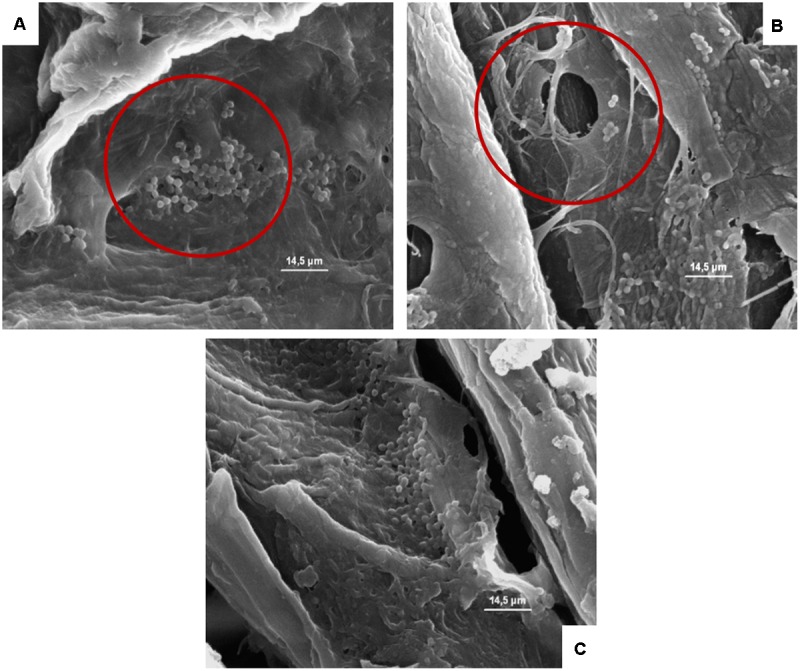
**(A–C)** Presence of the inoculated *L. monocytogenes* on cardboard surface after 1 h **(A)**, 24 h **(B)**, and 48 h **(C)**. In **A–C** the presence of pores and the entrapping of microbial cells between cardboard fibers are evidenced.

**FIGURE 8:**
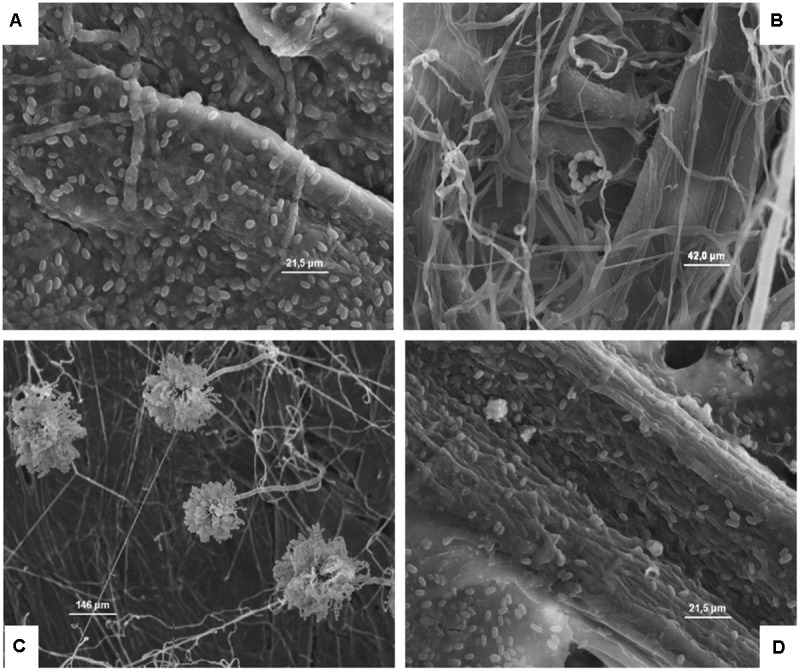
**(A–D)** Presence of the inoculated *A. flavus* on cardboard surface after 1 **(A)**, 24 **(B)**, and 48 h **(C)** in the presence of high relative humidity, and after 48 h in a dry environment **(D)**. The spores resulted not germinated after 1 h **(A)**, while after 24 and 48 h in the presence of high relative humidity a germination of the spore is clear and evidenced by the presence of mycelia and conidiophore **(B,C)**. By contrast, after 48 h in a dry environment no mycelia growth was observed.

**Figures 4–8**, relative to *Lb. plantarum*, *S. cerevisiae*, *E. coli*, *L. monocytogenes* vegetative cells, and *A. flavus* conidia after 1, 24, and 48 h, show the microorganisms entrapped within the cardboard fibers and pores. After 24–48 h, depending on the microbial species considered, the lyses also of the entrapped cells are evident. This means that also the entrapped cells, due to the lack of nutrient inside the cardboard fibers, die over time. This phenomenon is particularly evident in *S. cerevisiae* and *L. monocytogenes*. Also, the conidia of *A. flavus w*ere entrapped within the cardboard fibers. No mycelium growth was observed when the cardboard was completely dried after inoculation while with high relative humidity the growth of the mold mycelia was evident inside cardboard after 24 and 48 h. Since molds are important spoilage agents of fruits and vegetables, their proliferation on packaging material should be avoided in order to prevent the cross-contamination of the packaged fruits. Consequently, the storage of cardboard packaging materials at low relative humidity is fundamental to prevent the mold growth and the increase of their cell loads on surfaces in contact with fresh produces (Hladikova et al., 2015; Patrignani et al., 2016). The increase of the number of the cells entrapped within the corrugated fibers clearly demonstrates the reduction of the superficial contamination level and, consequently, the microbial capability to cross-contaminate the packed material.

Due to the compact structure (lacking pores able to entrap microorganisms) of plastic, the SEM analyses for this material were performed inoculating only *Lb. plantarum.* The results showed that the cells of this microorganism remained on the surface acting a major role in the cross-contamination of the packed products in accordance with the cell load data.

## Conclusion

The data clearly demonstrated that cardboard materials, if correctly stored, reduce the potential of packaging to the cross-contamination of food due to a quicker viability loss by spoilage and pathogenic microorganisms compared to the plastic ones. This phenomenon was observed for all the target microorganisms considered, except for molds which were able to proliferate when the absorption of water was not prevented during the storage. The SEM microphotographs showed the reduction of superficial contamination on cardboard surfaces was due to the entrapping of the microbial cells within the fibers and the pores of this packaging material and their death overtime due to the absence of water and nutrients. These results represent an objective evidence of previous findings on the reduced cross-contamination potential of corrugated compared with plastic packaging materials used in fruit and vegetable sector. Moreover, these results outlined the importance of storage and supply chain conditions (hygiene and low humidity) to prevent the mold growth and the increase of their role in the cross-contamination of packed foods.

## Author Contributions

LS, DS, and MB contributed to the acquisition, analysis, and interpretation of the data of the work. FP, CC, LG, and RL contributed to the design of the work, and the drafting and revision of the manuscript.

## Conflict of Interest Statement

The authors declare that the research was conducted in the absence of any commercial or financial relationships that could be construed as a potential conflict of interest.

## Footnotes

1 Recommendations for an environmental monitoring program for *Listeria monocytogenes* in processing areas. Guidelines on the Application of General Principles of Food.
